# Top-Down Reactive Approach for the Synthesis of Disordered ZrN Nanocrystalline Bulk Material from Solid Waste

**DOI:** 10.3390/nano10091826

**Published:** 2020-09-13

**Authors:** Mohamed Sherif El-Eskandarany, Sultan Majed Al-Salem, Naser Ali

**Affiliations:** 1 Energy and Building Research Center, Kuwait Institute for Scientific Research, Safat 13109, Kuwait; nmali@kisr.edu.kw; 2 Environment & Life Sciences Research Centre, Kuwait Institute for Scientific Research, Safat 13109, Kuwait; ssalem@kisr.edu.kw

**Keywords:** metal nitrides, refractory nanomaterials, reactive ball milling, spark plasma sintering

## Abstract

Transition metal nitrides possess superior mechanical, physical, and chemical properties that make them desirable materials for a broad range of applications. A prime example is zirconium nitride (ZrN), which can be obtained through different fabrication methods that require the applications of high temperature and pressure. The present work reports an interesting procedure for synthesizing disordered face centered cubic (fcc)-ZrN nanoparticles through the reactive ball milling (RBM) technique. One attractive point of this study is utilizing inexpensive solid-waste (SW) zirconium (Zr) rods as feedstock materials to fabricate ZrN nanopowders. The as-received SW Zr rods were chemically cleaned and activated, arc-melted, and then disintegrated into powders to obtain the starting Zr metal powders. The powders were charged and sealed under nitrogen gas using a pressurized milling steel vial. After 86 ks of milling, a single fcc-ZrN phase was obtained. This phase transformed into a metastable fcc-phase upon RBM for 259 ks. The disordered ZrN powders revealed good morphological characteristics of spherical shapes and ultrafine nanosize (3.5 nm). The synthetic ZrN nanopowders were consolidated through a spark plasma sintering (SPS) technique into nearly full-density (99.3% of the theoretical density for ZrN) pellets. SPS has proven to be an integral step in leading to desirable and controlled grain growth. Moreover, the sintered materials were not transformed into any other phase(s) upon consolidation at 1673 K. The results indicated that increasing the RBM time led to a significant decrease in the grain size of the ZrN powders. As a result, the microhardness of the consolidated samples was consequently improved with increasing RBM time.

## 1. Introduction

Research in advanced materials promotes development in societies [1]. Metallic glassy alloys, shape memory alloys, nanostructured materials, nanoparticles, nanocomposites, nanocrystalline refractory materials, conducting polymers, carbon nanotubes, nanodiamonds, graphene, bio-nanomaterials, 2-D nanomaterials, and high-entropy alloys are some examples. Refractory metal nitrides, in particularly group IV transition metals (TM; Ti, Zr, and Hf), have received great attention due to their outstanding properties. The interest in this group of TM nitrides that possesses mixtures of ionic, covalent, and metallic bonding is due to their desirable properties such as high thermal and excellent chemical stability, superior oxidation resistance, excellent thermal conductivity, high hardness, prime wear resistance, and excellent strength [2,3]. These properties makes them desired materials for various applications, particularly as a surface protective coating and in cutting tools [1,2,3].

For more than four decades, the cubic form of titanium nitride (TiN) has been the subject of research [4,5,6,7]. In contrast to TiN, the zirconium nitride (ZrN) binary system is relatively new and less studied. In addition to its applications in surface protective coatings and superhard cutting tools, ZrN has been implemented in photonics and plasmonics [8]. The ZrN system is typically prepared by different approaches, such as reduction–nitridation of zirconium oxide [3,9,10,11], reactive sputtering [12,13,14,15,16], chemical vapor deposition [17,18], self-propagating high-temperature [19], benzene-thermal synthesis [20,21], and physical vapor deposition (PVD) [22]. A downside of these methods is the fact that they require the application of high temperatures or pressures or both together. In the main process employed to produce industrial forms of ZrN, pure Zr metal and ZrCl_4_ are directly nitrided with ammonia gas at elevated temperatures (~1500 °C) [3]. The high cost of production alongside the presence of contaminants and the employment of environmentally unfriendly harmful gases are the main disadvantages of this method. The unfavorable aptness of the industrialized production of ZrN may lead to restriction of the mass production of such advanced material. Apart from the high-temperature/high-pressure approaches, the reactive ball milling (RBM) technique was introduced at the beginning of the 1990 s [23,24] and has been widely accepted for preparing metallic nitrides [25,26] and hydrides [27] at ambient temperature through high-energy ball milling. This process is a simplistic method that has proven its viability in such works many times over.

The present work introduces the preparation of nanocrystalline ZrN powders through the RBM process under pressurized nitrogen (N_2_) gas. Variations of RBM time are also reported. The present work also investigates the effect of grain size on the microhardness of spark plasma sintered (SPS) ZrN. A paramount advantage of this work is related to the use of solid waste (SW) Zr metal as a precursor material. The described RBM process in this work can be adapted and economically employed to produce nano-crystalline of ZrN powders and bulk materials at a large scale.

## 2. Materials and Methods

### 2.1. Feedstock Materials

In the present study, a batch of 1 kg of pure (99.0 wt%) scrap Zr rods (100–105 mm in length and ~10 mm in thickness) were acquired from Shanghai Xinglu Chemical Technology Co. (Pudong Dist, Shanghai, China) and used as feedstock materials. The received rods were cut into shorter ones (~50 mm in length) and then sonicated in a cold-acetone bath for 15 min to ensure the removal of all machining oil coolants from their surfaces. Rinsing of the rods took place with alcohol (pure ethanol) (Sigma-Aldrich Chemie GmbH, Eschenstrasse 5, 82024 Taufkirchen, Germany) and then they were dried for 16 h (200 °C). This primer treatment was conducted to remove the undesired hydrocarbon contaminants from the surfaces. An amount of 250 g of oil-free rods was then placed in the water-cooled copper hearth of an arc melter (Edmund Bühler GmbH, Hechingen, Germany), where the melting of Zr scrap was conducted using titanium (Ti), under less than 0.8 bar of pure argon gas (Ar). The molten Zr button was turned over and remelted 5 times. The chemical analysis of the obtained Zr button showed that it possessed high purity of 98.7 wt%, with 0.8, 0.3, and 0.2 wt%, respectively, of hafnium (Hf), oxygen (O_2_), and carbon (C).

### 2.2. Sample Preparations

The Zr button was crushed down into small pieces of almost 2.5 cm in size and was charged into a Model RS 200 vibratory disc mill machine (Retsch GmbH Company, 42781 Haan, Germany) operated at a rotation speed of 1500 rpm for 2 min. Powders were sieved to obtain a product of less than 50 μm in diameter. Powders were placed in a helium UNILAB Pro Glove Box Workstation (M. BRAUN INERTGAS-SYSTEME GMBH, Garching, Germany) sealed with 50 balls of 11 mm (dia) tool steel balls coupled with an evico magnetics gas monitoring system (Mettler-Toledo GmbH, Ockerweg Giessen, Germany) (40:1 ball to powder). The process was continuously operated until selected times were reached to obtain 300 mg of powder using a Fritsch PULVERISETTE 6 mill (FRITSCH GmbH, Broyage et Granulométrie Idar-Oberstein, Germany).

### 2.3. Powder Consolidation

Powders which were as-ball milled with 11, 22, 43, 85, 175, 259, and 360 ks of continuous RBM were individually consolidated into dense buttons through spark plasma sintering (SPS) acquired from Dr. Sinter Lab. Instrument Co. Tokyo, Japan. The system consists of a press with vertical single-axis pressurization, electrodes incorporating a water cooler, a water-cooled vacuum chamber, a vacuum/air/argon-gas atmosphere control mechanism, a special direct current (DC) pulse sintering power generator, a cooling-water control unit, a Z-axis position measuring and control unit, temperature measuring and control units, an applied pressure display unit, and various safety interlock devices. In this study, powders obtained were placed into a die made of graphite. Sheets of graphite were also used to avoid reactions between surfaces. The die as a whole was wrapped with carbon felt held with a carbon yard in an effort to reduce heat transfer. An electric field was used to control the sintering. Rather than hot-pressing [25], in this work we used sintering SPS conducted by internally heating samples by electric current passage. Heating and cooling rates of 580 and 280 K/min were used, respectively. An external pressure was applied during sintering in the range of 10–15 MPa (1673 °C). The process as a whole took about 6 min.

### 2.4. Sample Characterizations

#### 2.4.1. Crystal Structure

CuKα radiation X-ray diffraction (XRD) analysis was conducted using 9 kW SmartLab-Rigaku XRD system (Applied Rigaku Technologies, Inc., Austin, TX 78717 USA). Field emission high resolution transmission electron microscopy (HRTEM) coupled with scanning transmission electron microscopy (STEM) was also used by operating a JEOL-2100F equipped Oxford Instruments EDS (Oxford Instruments NanoAnalysis & Asylum Research, UK). The objective lens spherical aberration coefficient (Cs) of this microscope is 0.5 mm, where the point resolution is 0.19 nm, and the lattice resolution is 0.12 nm. The maximum and minimum spot sizes for the nano beam diffraction (NBD) were 0.5 and 25 nm, respectively. A Cryo Ion IB-09060CIS slicer machine (JEOL, Musashino, Akishima, Tokyo, Japan) was also used to prepare the TEM specimens as a consolidated ZrN.

#### 2.4.2. Morphology and Elemental Analysis

Field emission microscopy (FE-SEM) was used to study the samples with a 15 kV voltage (JSM-7800F JEOL Co., Musashino, Akishima, Tokyo, Japan), and elemental analysis (EA) was also conducted with an Oxford Co., UK, EDS interface (Oxford Instruments NanoAnalysis & Asylum Research, UK).

#### 2.4.3. Density and Microhardness

The density was determined using toluene media. A 1 kg quantity of Vickers indenter was used to determine the microhardness of the compacted samples with an average reading of 10 indentations.

## 3. Results

### 3.1. Crystal Structure and Morphology

**Figure 1:**
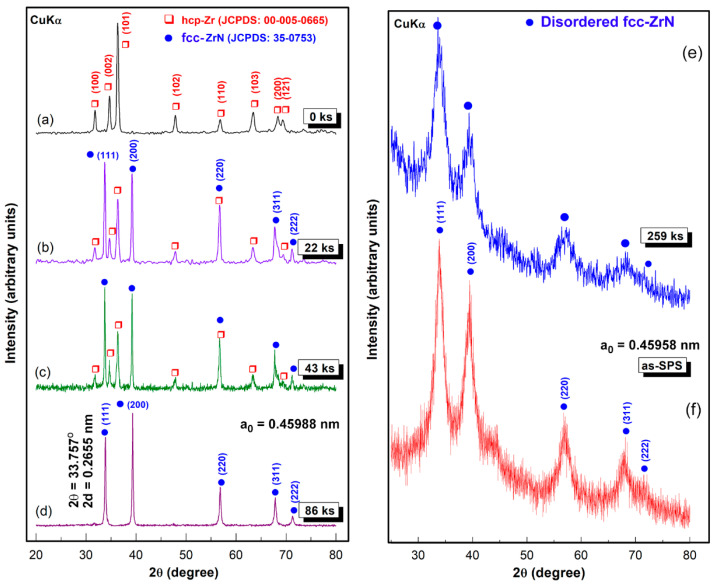
X-ray diffraction (XRD) patterns of hcp-Zr metallic powder that were high-energy ball-milled for (**a**) 0 ks; (**b**) 22 ks; (**c**) 43 ks; (**d**) 86 ks; and (**e**) 259 ks of the reactive ball milling (RBM) time, (**f**) XRD pattern of the spark plasma sintered (SPS) 259 ks-powders.

**Figure 2:**
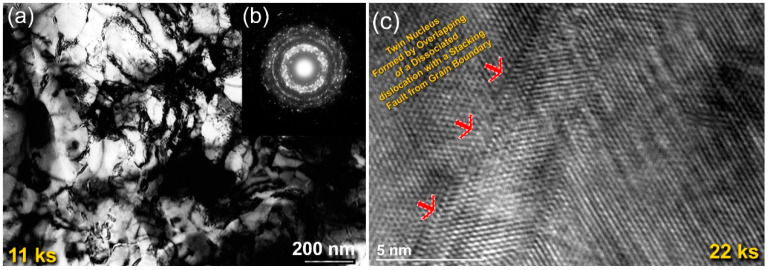
(**a**) Bright-field (BF) image; (**b**) the corresponding selected area diffraction pattern (SADP) of Zr powders obtained after 11 ks of RBM time; (**c**) field-emission scanning electron microscope (FE-HRTEM) image of the RBM powders for 22 ks.

**Figure 3:**
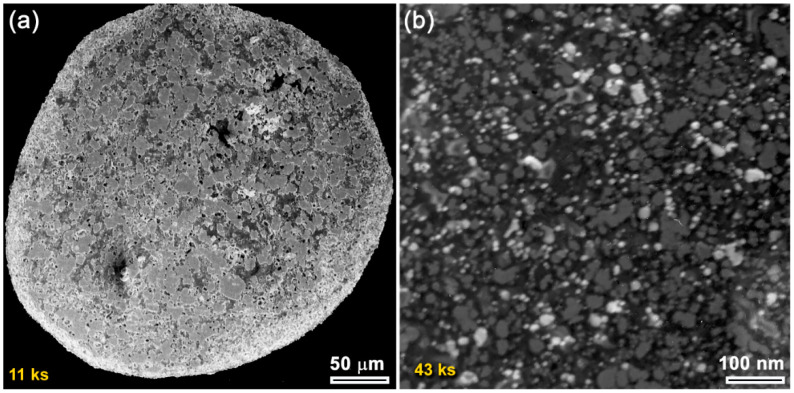
(**a**) Field-emission scanning electron microscope (FE-SEM) micrograph of Zr powders obtained after 11 ks of RBM time; (**b**) scanning transmission electron microscopy (STEM) image of as-RBM powders for 43 ks.

Progress of the mechanically-induced gas-solid reaction conducted by the RBM process was monitored through XRD and HRTEM analysis. A sharp Bragg-peak that corresponded to hexagonal closest packed (hcp)-Zr metal (JCPDS: 00-005-0665) was noticed initially. There was also an absence of other phases (Figure 1a). During the first few kiloseconds (11 ks) of RBM, the Zr powders of this milling stage were composed of large grains in the range from 65 nm to 380 nm (Figure 2a). The corresponding area from the middle zone of Figure 2a showed sharp spot-like diffraction (Figure 2b), implying the existence of microscaled grains of Zr. The increase in the RBM time (22 ks) led to the enhancement of the mechanical deformation in the Zr lattice, as implied by the massive imperfections of stacking faults beyond the atomic level that were overlapped with extrinsic dislocations and nano twins (Figure 2c). Meanwhile, the powders at this stage were shattered and disintegrated to create large spherical Zr particles with very clean oxygen-free active surfaces, as displayed in Figure 3a. The formation of the fcc-ZrN phase with a space group of Fm3$\bar{m}$ [225] was confirmed with an analysis of the new Bragg-lines ((111), (200), (220), (311), and (222)) that were detected in Figure 1b. The results affirmed an excellent match with the reference data reported in JCPDS 35-0753.

At this early stage of RBM, the reacted ZrN phase still coexisted with unprocessed hcp-Zr powders, as presented in Figure 1b. As the RBM time increased (43 ks), the Zr powders were fragmented into smaller active-surface particles with a large surface area. Therefore, the Bragg-peak intensity related to fcc-ZrN became pronounced, where the Bragg-peak intensity of pure Zr was inclined to decrease (Figure 1c). Thereby, an increase in the molar fraction of ZrN against the unprocessed Zr metal powders was attained. The scanning transmission electron microscope (STEM) images of the milled powders for 43 ks of RBM time signified the formation of aggregated nanopowders (~10 nm), as shown in Figure 3b.

After 86 ks of RBM time, all the Bragg-peaks related to unprocessed hcp-Zr powders had entirely disappeared, where a single phase of fcc-ZrN was present (Figure 1d), implying the completion of the RBM process. The lattice parameter (a_0_) of the as-prepared ZrN phase was calculated from the (111) reflection and found to be 0.45988 nm, which was in fair agreement with the available reference (JCPDS 35-0753). The useful kinetic energy in a high-energy ball mill is generated by the following means: (1) collision between the balls and the particle; (2) pressure loading of particles pinned between milling media or between the milling media and the liner; (3) impact of the falling milling media; (4) shear and abrasion caused by dragging of particles between moving milling media; and (5) shock-wave transmitted through crop load by falling milling media.

**Figure 4:**
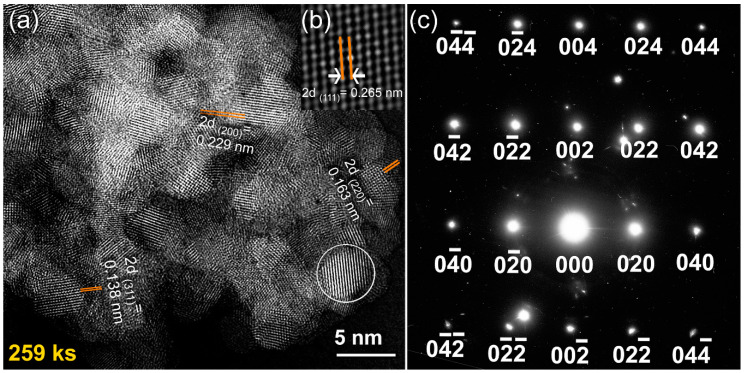
(**a**,**b**) FE-HRTEM dark field (DF) images; (**c**) the corresponding nanobeam diffraction patterns (NBDP) pattern of ZrN powders obtained after 259 ks of the RBM time.

The FE-HRTEM dark field (DF) image for this end-product confirmed the formation of nanocrystalline fcc-ZrN grains (~3.5 nm) that did not reveal a specific orientation, as elucidated in Figure 4a. The dislocations existed in the individual ZrN grains suggesting the nanostructure development attained by severe plastic deformation upon the RBM process. Due to the increased volume fraction of the ultrafine grains, a large number of grain boundaries (GB) were developed. The fringe atomic-resolution TEM image of a single ZrN grain located in Zone I is displayed in Figure 4b. Using FE-HRTEM with an objective lens spherical aberration coefficient (Cs) of 0.5 mm, point resolution of 0.19 nm, and lattice resolution of 0.12 nm, the interplanar spacing (2d) elucidated in Figure 4b was measured and found to be 0.265 nm, matching well with ZrN (111). Moreover, the Miller indexed nanobeam diffraction pattern (NBDP), using a NBD spot size of 0.5 nm, which corresponding to Zone I, revealed the fcc-structure in the zone axis direction of [100], which evidenced the disordered formation the fcc-ZrN phase (Figure 4c).

### 3.2. Consolidation of Disordered fcc-ZrN

**Figure 5:**
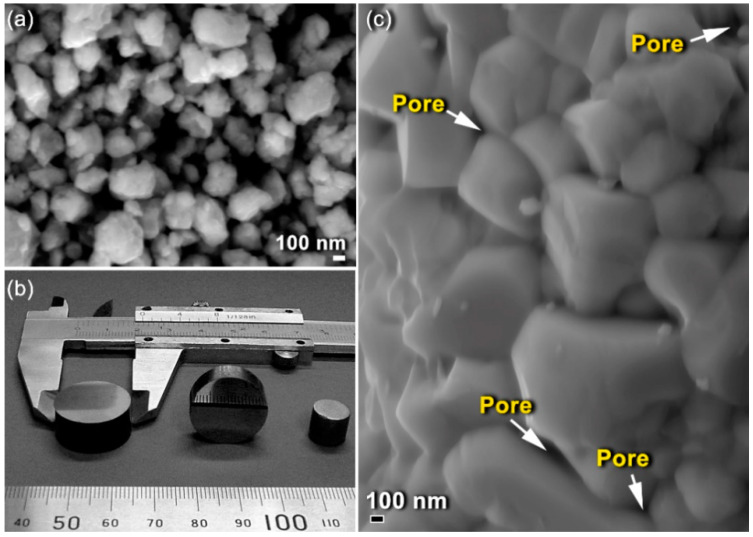
(**a**) Field-emission scanning electron microscope (FE-SEM) micrograph of fcc-ZrN powders obtained after 259 ks of RBM; (**b**) the outer shape view of the as-consolidated powders through SPS; (**c**) surface fracture of the consolidated sample.

The end-product of fcc-ZrN nanopowders, obtained after 259 ks of RBM time, which was composed of nanosized grains of ~3.5 nm (Figure 4a), was consolidated through the SPS technique into nearly-net shape dense objects (Figure 5). The as-SPS consolidated buttons had a uniform cylindrical shape and exhibited a high-density appearance without indications of the existence of any cracks or pores, as shown in Figure 5. The bulk density of the as-consolidated samples was measured via Archimedes’ principle using water immersion and found to range between 7.29 g/cm^3^ and 7.31 g/cm^3^. Comparing these values with the theoretical density for ZrN (7.35 g/cm^3^) indicates that the consolidated fcc-ZrN samples were nearly fully dense. The SPS procedure did not lead to a dramatic grain growth or phase transformation, as characterized by the broadening of the ZrN Bragg-peaks (Figure 1f). The consolidation of nanocrystalline ZrN powders into full-density coherent bulk objects with minimal particle coarsening and grain growth many challenges attributed to the large surface area and great volume fractions of GBs for the as-prepared ZrN powders. Accordingly, they were highly unstable and could undergo undesired grain growth when subjected to high temperatures.

**Figure 6:**
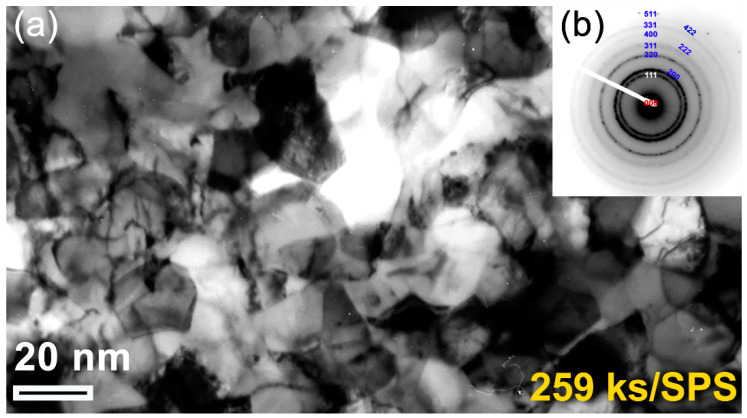
(**a**) Bright field image (BFI) of the planer view for the as-consolidated fcc-ZrN metastable phase that was obtained after 259 ks of RBM and then SPS; (**b**) the related SADP, which was taken from the middle zone of (**a**).

The FE-HRTEM analysis indicated that the SPS process led to slight grain growth of ZrN powders, as implied by the enlarged ZrN grains obtained after consolidation, which were found to be in the range from 6.3 nm to 26.5 nm (Figure 6). Comparing these values with the originally milled powders (~3.5 nm), we can claim that SPS may lead to moderate grain growth. Moreover, the selected area diffraction pattern (SADP) related to Figure 6a revealed simultaneously diffracted Debye–Scherrer rings, which were indexed to fcc-ZrN. The absence of any other diffracted rings and sharp spots suggested the formation of nanocrystalline fcc-ZrN, as displayed in Figure 6b. We should clarify that the as-prepared nanocrystalline ZrN powders had numerous volume fractions of GBs (Figure 4a). Such GBs possess high-energy and a thermodynamic force to reduce the overall area of GBs through grain coarsening. This makes the nanograins ZrN powders intrinsically unstable.

**Figure 7:**
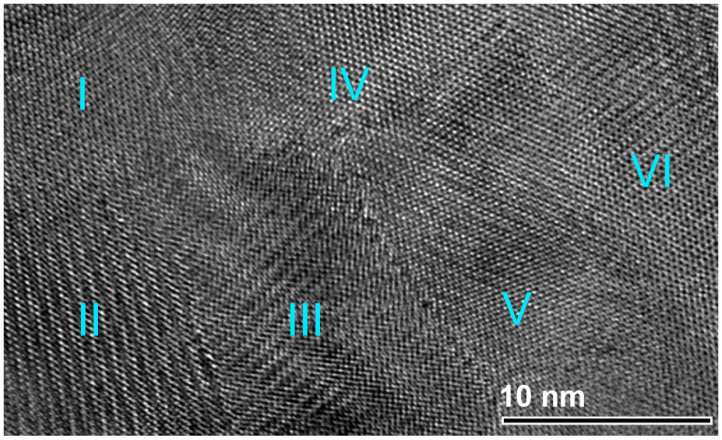
An atomic-resolution FE-TEM of the planer view for the as-consolidated fcc-ZrN metastable phase that was obtained after 259 ks of RBM and then SPS. The image, which presents six (I to VI) nanograins, indicates a highly-dense structure, and the existence of stacking faults and twin boundaries.

In this study, we attempted to overcome the thermal coarsening of the grains through a primer annealing step of the powders inside the SPS graphite sample holder. This preliminary thermal treatment before consolidation led us to deduce the grain growth of ZrN powders. The in-situ post-annealing step had beneficial consequences for the formation of a nanograin structure with a high-density of coherent twin boundaries, which are low energy, and low mobility boundaries with a high degree of crystallographic ordering, as confirmed by FE-HRTEM analysis (Figure 7). Accordingly, preliminary thermal treatment would provide the required resistance to the thermal coarsening during consolidation.

## 4. Discussion

**Figure 8:**
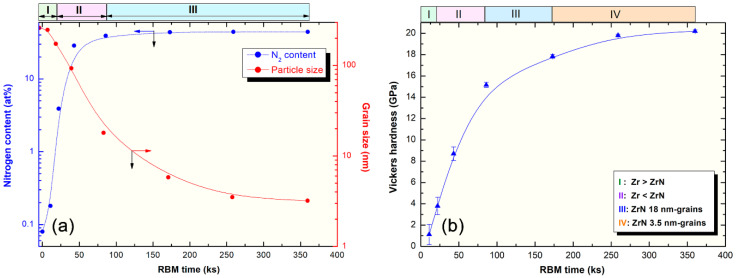
(**a**) Effect of the RBM time on the grain size, and nitrogen content upon high-energy milling of hcp-Zr metal under 70 bar nitrogen gas pressure; (**b**) influence of RBM time on the microhardness of ZrN consolidated buttons.

Despite the traditional approaches used for preparations of metal nitrides under the applications of high temperature and pressure, the RBM technique has been utilized to synthesize different families of nanocrystalline metal nitrides. In the present study, a single phase of metastable fcc-ZrN nanopowders was prepared through high-energy RBM at room temperature. This process can be classified into three consequent stages, as elucidated in Figure 8.

In the first stage (0 ks–22 ks) of the RBM, the starting Zr powders were subjected to GB defects (Figure 2a), excessive plastic deformation (Figure 2c), and lattice imperfections (e.g., dislocations, point defect, and vacancies). These structural-deformation defects, which were localized in the shear bands of Zr, led to the introduction of a highly dense network of dislocations, as can be noted in Figure 2a. These localized severe defects stimulated the instabilities of the Zr lattice. Moreover, the atomic level strain was remarkably increased as a result of expanding the dislocation density in the overall hcp-Zr powders. Due to these successive accumulations of the dislocation density generated during the second stage of RBM (22 ks–86 ks), the large grains of starting Zr powders (~260 nm) were disintegrated into finer subgrains grains of ~173 nm after 22 ks of RBM time, as presented in Figure 8. The defragmentation of microscaled-Zr grains (Figure 3a) into nanosized grains (~90 nm) after 43 ks of RBM time (Figure 3b) can be attributed to a gradual decrease in the atomic strain. The Zr powders were, therefore, disintegrated into smaller particles with large surface area, and very clean oxygen-free active surfaces. Accordingly, the reactive milling atmosphere of nitrogen was guttered and absorbed completely by the first atomically clean surfaces of Zr powders. This could be realized by the dramatic increase of nitrogen absorbed by Zr powders (Figure 8a).

We should emphasize that the diffusion of nitrogen in the Zr powders was enhanced upon the existence of crystal defects in the Zr lattice. In other words, increasing the volume fraction of the nanosized grains upon introduction of GB defects led to improvement in the solubility of nitrogen in Zr. For instance, when Zr grains were 173 nm, the powders were not able to absorb more than 4 wt% of nitrogen (Figure 8a). The reduction of the grain size (93 nm) attained after 43 ks of the RBM time improved the uptake capability of Zr powders to absorb almost 30 wt% of nitrogen, as demonstrated in Figure 8. It can be claimed, therefore, that GB defects may provide an excellent pathway for rapid diffusion of nitrogen. After 86 ks, a single phase of fcc-ZrN was obtained, as shown in Figure 1d and Figure 4b. This stoichiometric phase (~39 wt% nitrogen) could no longer withstand the lattice imperfections generated by the ball milling media, where it tended to transform into a metastable phase of fcc-ZrNi, as displayed in Figure 1e. During the last stage of the RBM (86 ks–360 ks), the brittle ZrN powders were dramatically disintegrated into ultrafine grains with sizes in the range from 3.5 nm to 5 nm, as presented in Figure 4a. The corresponding increase of nitrogen content in this stage of RBM (Figure 8a) may be attributed to a reaction conducted between ultrafine Zr powders that existed in the end product of the fcc-ZrN.

The present work reports sintering with SPS that is commonly known as the field-assisted sintering technique (FAST), as reported in the methods section. Pressure was externally applied (10–15 MPa). Consolidation was achieved by charging the intervals between the particles of the powders with electrical energy and effectively applying spark plasma. One merit of the SPS consolidation step is that it maintains the original nanocrystalline grains without dramatic growth (Figure 8a). The variation of Vickers microhardness (VMH) with RBM time of as-SPS samples is displayed in Figure 8b. The influence of RBM time on VMH values can be realized. This monotonical increase is attributed to the consequent decrease of the grain size upon increasing RBM time (Figure 8a). After 11 ks of RBM time, VMH revealed a wide distribution in the range from 0.15 GPa to 2 GPa, as shown in Figure 8b. The VMH of the as-SPS sample of the powders milled for 22 ks was varied from 3 GPa to 4.6 GPa. This significant broad variation decreased with increasing RBM time (43 ks) to be in the range of 8 GPa to 9.4 GPa, as presented in Figure 8b. This significant broad variation in VMH is attributed to the existence of a large volume fraction of unprocessed Zr in the original feedstock of RBM powders, as evidenced by the XRD analysis (Figure 1b,c). The VMH of the SPS sample obtained of the powders milled for 86 ks was 15.14 GPa, as shown in Figure 8b. The dramatic increase in VMH of this sample suggested the absence of unprocessed Zr metal, where a single fcc-ZrN phase existed (Figure 1d).

Further RBM time (86 ks–360 ks) led to the refining of the ZrN powders, which was necessary to obtain ultrafine powders of ZrN nanograins, as demonstrated in Figure 8a. During this final stage of RBM, the VMH increased significantly to attain higher values of 17.84 GPa and 19.8 GPa after RBM times of 173 ks and 259 ks, respectively (Figure 8b). As the grain size of the consolidated 360 ks-ZrN sample did not show a remarkable change (Figure 8a), the VMH tended to be saturated at 20.2 GPa, as displayed in Figure 8b. The grain size softening effect concerning the hardness (inverse Hall–Petch) is an accepted phenomenon used to interpret the increase of hardness upon grain size reduction for nanocrystalline materials. This beneficial effect is attributed to the changes in the plastic deformation mechanisms, as pointed out by several authors. In general, low-temperature plastic deformation during the Vickers hardness test is principally controlled by the dynamics of dislocations in the well-ordered structure of the grain interiors. The dislocation slip taking place within the deformation is blocked by the grain boundaries, which act as deformation barriers. As a result, the hardness increases with decreasing grain size, as described in the Hall–Petch relationship, over a wide range of grain size, as presented in Figure 8a,b. It should be noted that the VMH value of SPS-fcc-ZrN nanocrystalline metastable phase (~20 GPa) of this work is far above that of the regular polycrystalline ZrN (15.8 GPa).

## 5. Conclusions

Utilization of solid waste (SW) Zr metal for tailoring nanocrystalline ZrN nanoparticles has not yet been reported. The present study demonstrated an effective multi-stage process used to fabricate high-grade Zr metal starting from recycled Zr rods. Chemical treatment and arc melting were dedicated to producing Zr buttons (99.88 wt%). The as-disintegrated arc melt Zr button was used as feedstock materials for synthesizing nanocrystalline ZrN powders through a reactive ball milling (RBM) approach under pressurized nitrogen gas. A single fcc-ZrN phase was obtained upon RBM for 86 ks. This phase could not withstand the mechanical deformation generated by the milling media and transformed into a metastable fcc-phase after 259 ks of RBM. The as-prepared ZrN disorder powders possessed ultrafine nanocrystalline structures composed of spherical grains of ~3.5 nm. The end product of ZrN nanoparticles maintained their nano characteristics with nearly full density (~99.3%) buttons when consolidated through a spark plasma sintering (SPS) technique at 1673 K under 10–15 MPa. The SPS procedure did not cause undesired grain growth and/or phase separation. Additionally, the nanocrystalline fcc-ZrN disordered phase even maintained its metastability and did not tend to transform into any other phase(s) after SPS.
